# Nicotine and sleep deprivation: impact on pain sensitivity and immune modulation in rats

**DOI:** 10.1038/s41598-018-32276-7

**Published:** 2018-09-14

**Authors:** Camila Hirotsu, Matheus Negrao Pedroni, Laís Fernanda Berro, Sergio Tufik, Monica Levy Andersen

**Affiliations:** 1 Department of Psychobiology, Universidade Federal de São Paulo, São Paulo, Brazil; 2 Department of Psychiatry and Human Behavior, University of Mississippi Medical Center, Jackson, USA

## Abstract

Repeated nicotine administration has been associated with increased paradoxical sleep in rats and antinociceptive properties, whereas paradoxical sleep deprivation (PSD) elicits pronociceptive and inflammatory responses. Thus, we aimed to evaluate the effect of repeated nicotine administration and its withdrawal combined with PSD on pain sensitivity and inflammatory markers. Sixty adult male Wistar rats were subjected to repeated injections of saline (SAL) or nicotine (NIC) for 12 days or 7 days of nicotine followed by acute mecamylamine administration on day 8 to precipitate nicotine abstinence (ABST). On day 9, the animals were submitted to PSD for 72 h or remained in control condition (CTRL); on day 12, thermal pain threshold was assessed by the hot plate test. PSD significantly decreased the latency to paw withdrawal in all groups compared to their respective controls. ABST-PSD animals presented higher levels of interleukin (IL)-6 compared to all groups, except ABST-CTRL. After adjustment for weight loss, IL-6, IL-4 and tumor necrosis factor alpha, ABST-PSD was associated with the lowest pain threshold. Nicotine and IL-4 levels were predictors of higher pain threshold. Hyperalgesia induced by PSD prevailed over the antinociceptive action of nicotine, while the association between PSD and ABST synergistically increased IL-6 concentrations and decreased pain threshold.

## Introduction

As a stimulant of the central nervous system, nicotine may affect sleep pattern in both humans and rodents^1,2^. In nonsmokers, acute nicotine administration has been associated with a dose-dependent reduction of rapid eye movement (REM) sleep and slow-wave sleep^3,4^. When in nicotine abstinence, a significant increase in total sleep time and REM sleep rebound occur^3^. In rats, acute nicotine administration has also been reported to decrease both paradoxical sleep and slow-wave sleep in a dose-dependent manner^2^. Nevertheless, during repeated administration, nicotine leads to an increase of paradoxical sleep^2^, possibly mediated by neuronal nicotinic acetylcholine receptors (nAChR) in the pontine reticular formation of rats^5,6^. Moreover, after mecamylamine-induced withdrawal, nicotine-treated animals display a normal sleep pattern^2^.

Animal models of chronic pain present decreased percentage of time in paradoxical sleep, suggesting an important role of REM sleep in pain regulation^7,8^. Conversely, paradoxical sleep deprivation (PSD) may elicit higher pain sensitivity in rodents^9,10^. On the other hand, both systemic and intrathecal administrations of nicotine have demonstrated antinociceptive properties in different animal models of pain^6,11,12^ as well as anti-inflammatory properties^13–15^.

Considering that nicotine and PSD exert opposed effects on nociception, this work aimed to evaluate the effects of repeated nicotine administration and its withdrawal combined with PSD on pain sensitivity. We hypothesized that chronic nicotine treatment would at least partially counteract the effects of PSD on pain sensitivity in association with higher anti-inflammatory and lower pro-inflammatory cytokines levels. In addition, we expected that during nicotine withdrawal, PSD would lead to the highest levels of pain sensitivity and pro-inflammatory cytokines. Secondarily, we aimed to investigate the independent predictors of pain threshold.

## Results

### Body Weight Loss due to Sleep deprivation

**Figure 1:**
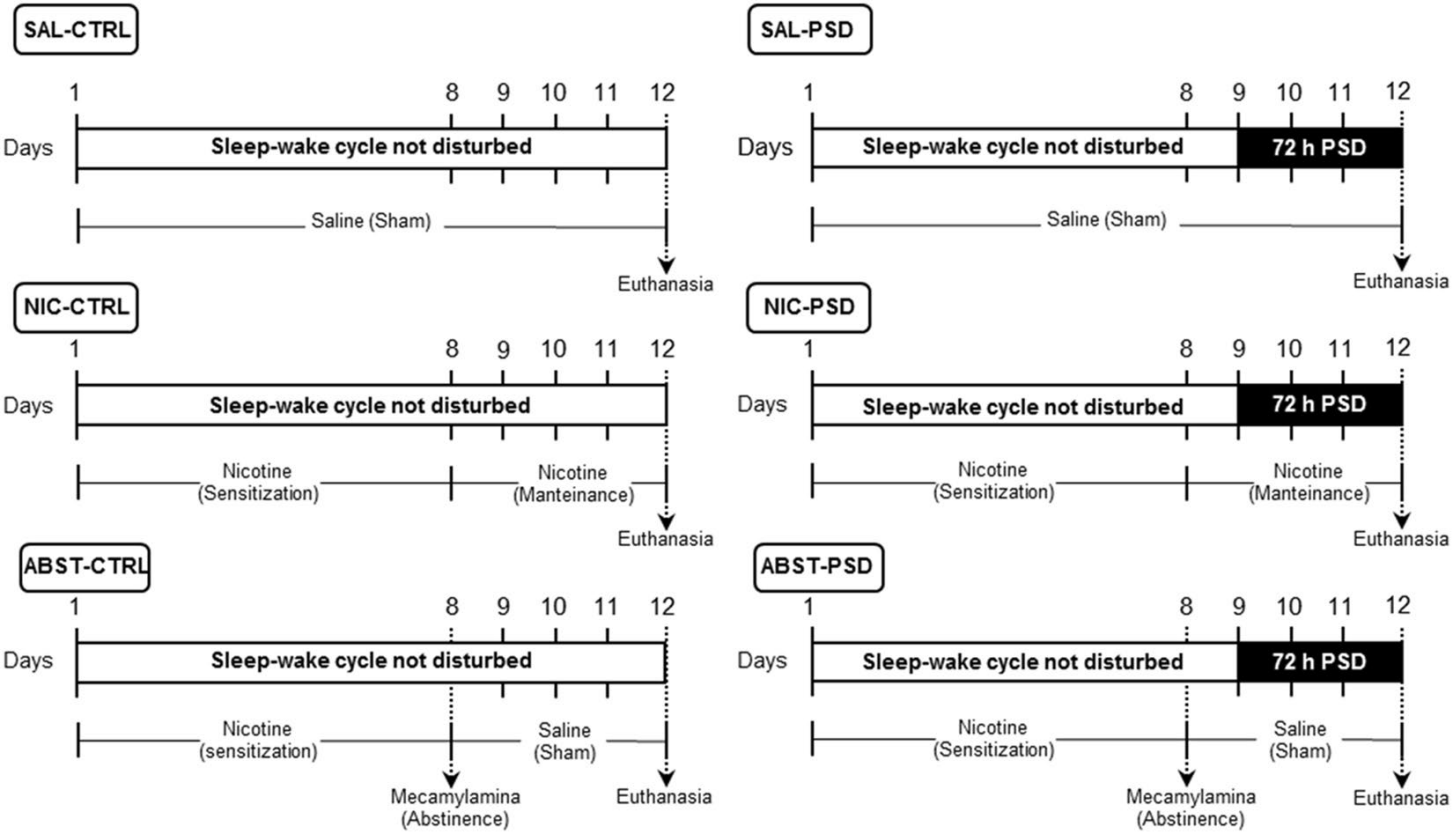
Experimental protocol. Experimental timeline for each condition: SAL-CTRL (saline treatment followed by control sleep condition, n = 10), NIC-CTRL (nicotine sensitization followed by control sleep condition, n = 10), ABST-CTRL (nicotine sensitization and mecamylamine-induced withdrawal followed by control sleep condition, n = 10), SAL-PSD (saline treatment followed by 72 h of paradoxical sleep deprivation (PSD), n = 10), NIC-PSD (nicotine sensitization followed by 72 h of PSD, n = 10), ABST-PSD (nicotine sensitization and mecamylamine-induced withdrawal followed by 72 h of PSD, n = 10).

**Figure 2:**
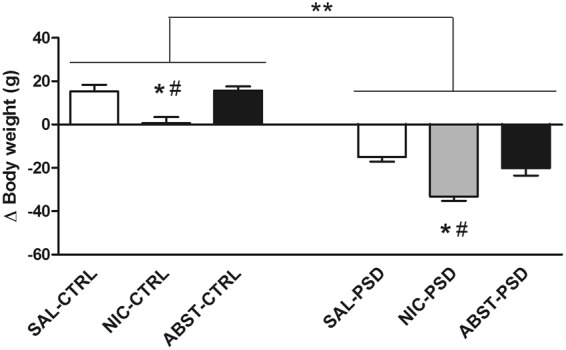
Body weight. Difference between final (Day 12) and initial (Day 1) body weight in animals submitted to a 12-day saline (SAL) treatment, a 12-day nicotine (NIC, 3.2 mg/kg/day) treatment or a 7-day NIC treatment followed by mecamylamine-induced withdrawal (1.5 mg/kg) and subsequently exposed to control sleep condition (CTRL) or 72 h paradoxical sleep deprivation (PSD) (n = 10 per group). *p < 0.0001 compared to the respective SAL group (CTRL or PSD); ^#^p < 0.0001 compared to the respective ABST group (CTRL or PSD); **p < 0.0001 significant effect of PSD.

Animals were either treated with saline (SAL), nicotine for 12 days (NIC) or nicotine for seven days followed by an injection of mecamylamine to precipitate nicotine withdrawal and five more days of saline treatment (ABST). On day 9, the animals were distributed into two conditions: normal sleep (CTRL) or 72 h of PSD, thus resulting in six experimental groups. The experimental protocol is represented in Fig. 1. The delta body weight (the difference between body weight at day 12 and day 1) for each group is shown in Fig. 2. PSD groups had a significant body weight loss in comparison to the CTRL groups (F_1,54_ = 236.4, p < 0.0001), independent of nicotine treatment. Additionally, a treatment effect was observed (F_2,54_ = 22.5, p < 0.0001), revealing that NIC animals had lower body weight gain compared to both SAL (p < 0.0001) and ABST groups (p < 0.0001). Additionally, a *post-hoc* test of the univariate analysis showed that among CTRL groups, delta body weight was lower only in NIC-CTRL compared to both SAL-CTRL (p = 0.001) and ABST-CTRL (p < 0.0001) groups. Similarly, NIC-PSD group presented lower delta body weight compared to both SAL-PSD (p < 0.0001) and ABST-PSD (p = 0.003) groups.

### Sleep Deprivation Overcame the Analgesic Effect of Nicotine

**Figure 3:**
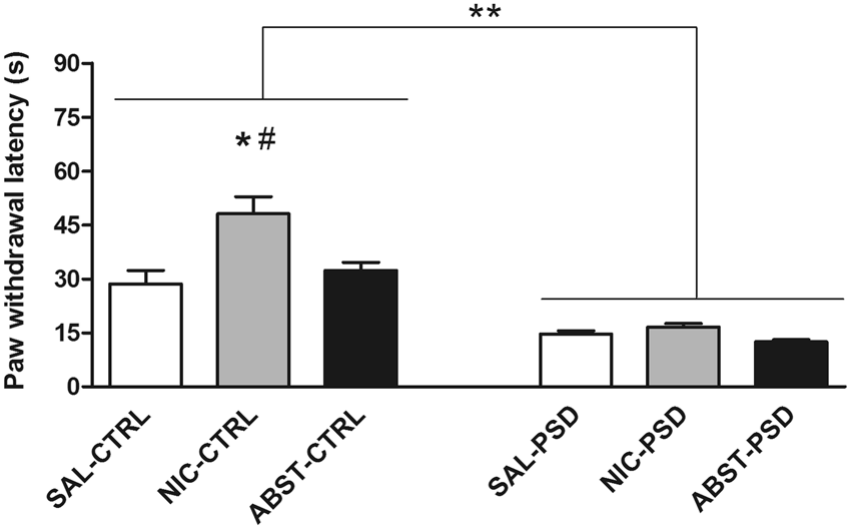
Pain sensitivity. Mean ± standard deviation of latency to paw withdrawal (s) in the hot plate test in animals submitted to a 12-day saline (SAL) treatment, a 12-day nicotine (NIC, 3.2 mg/kg/day) treatment or a 7-day NIC treatment followed by mecamylamine-induced withdrawal (1.5 mg/kg) and subsequently exposed to control sleep condition (CTRL) or 72 h paradoxical sleep deprivation (PSD) (n = 10 per group). *p < 0.001 compared to SAL-CTRL; ^#^p < 0.0001 compared to ABST-CTRL; **p < 0.05 significant effect of PSD.

Nociceptive sensitivity was assessed by the hot plate test, in which the animal is placed over a heated plate and the latency to paw withdrawal is used as a measure of pain threshold and sensitivity. This result is shown in Fig. 3. When not adjusted for any confounder, the analysis of the latency to paw withdrawal demonstrated treatment (F_2,54_ = 9.4, p < 0.0001), PSD (F_1,54_ = 93.2, p < 0.0001) and interaction effects (F_2,54_ = 5.2, p < 0.01). Overall, NIC animals showed a higher latency to paw withdrawal when compared to both SAL (p < 0.0001) and ABST groups (p < 0.0001). Alternatively, PSD led to a shorter latency in all the three groups (SAL-PSD, NIC-PSD and ABST-PSD) compared to SAL-CTRL (p < 0.0001), NIC-CTRL (p < 0.0001) and ABST-CTRL (p < 0.0001), respectively. However, a *post-hoc* test of the interaction effect revealed that only the NIC-CTRL group had a significant increase in pain threshold compared to SAL-CTRL (p < 0.0001), SAL-PSD (p < 0.0001), NIC-PSD (p < 0.0001), ABST-CTRL (p = 0.005), and ABST-PSD (p < 0.0001) groups. No significant changes in pain sensitivity were observed among the PSD groups (SAL-PSD, NIC-PSD, and ABST-PSD).

After adjusting for potential confounders or mediators (delta body weight; tumor necrosis factor alpha, TNF-α; interleukin 4, IL-4; and IL-6), we also observed treatment (F_2,54_ = 24.2, p < 0.0001), PSD (F_1,54_ = 33.0, p < 0.0001) and interaction effects (F_2,54_ = 13.7, p = 0.001). However, *post-hoc* tests of interaction effect showed that PSD significantly decreased pain threshold only in NIC-PSD (18.1 ± 7.7 s) and ABST-PSD (11.0 ± 3.8 s) groups compared to NIC-CTRL (49.8 ± 10.9 s, p < 0.0001) and ABST-CTRL (30.1 ± 10.4 s, p < 0.0001), respectively. No significant changes were observed between SAL-PSD (16.3 ± 4.9 s) and SAL-CTRL (26.1 ± 9.4, p = 0.142). As in the unadjusted analysis, NIC-CTRL presented higher pain threshold compared to all other groups (p < 0.0001). Additionally, ABST-PSD exhibited the lowest pain threshold, which statistically differed from SAL-CTRL (p < 0.0001), NIC-CTRL (p < 0.0001), ABST-CTRL (p < 0.0001) and NIC-PSD (p = 0.040) groups, with a trend of difference compared to SAL-PSD (p = 0.082).

### Sleep Deprivation and Nicotine Abstinence Increased IL-6 concentrations

**Figure 4:**
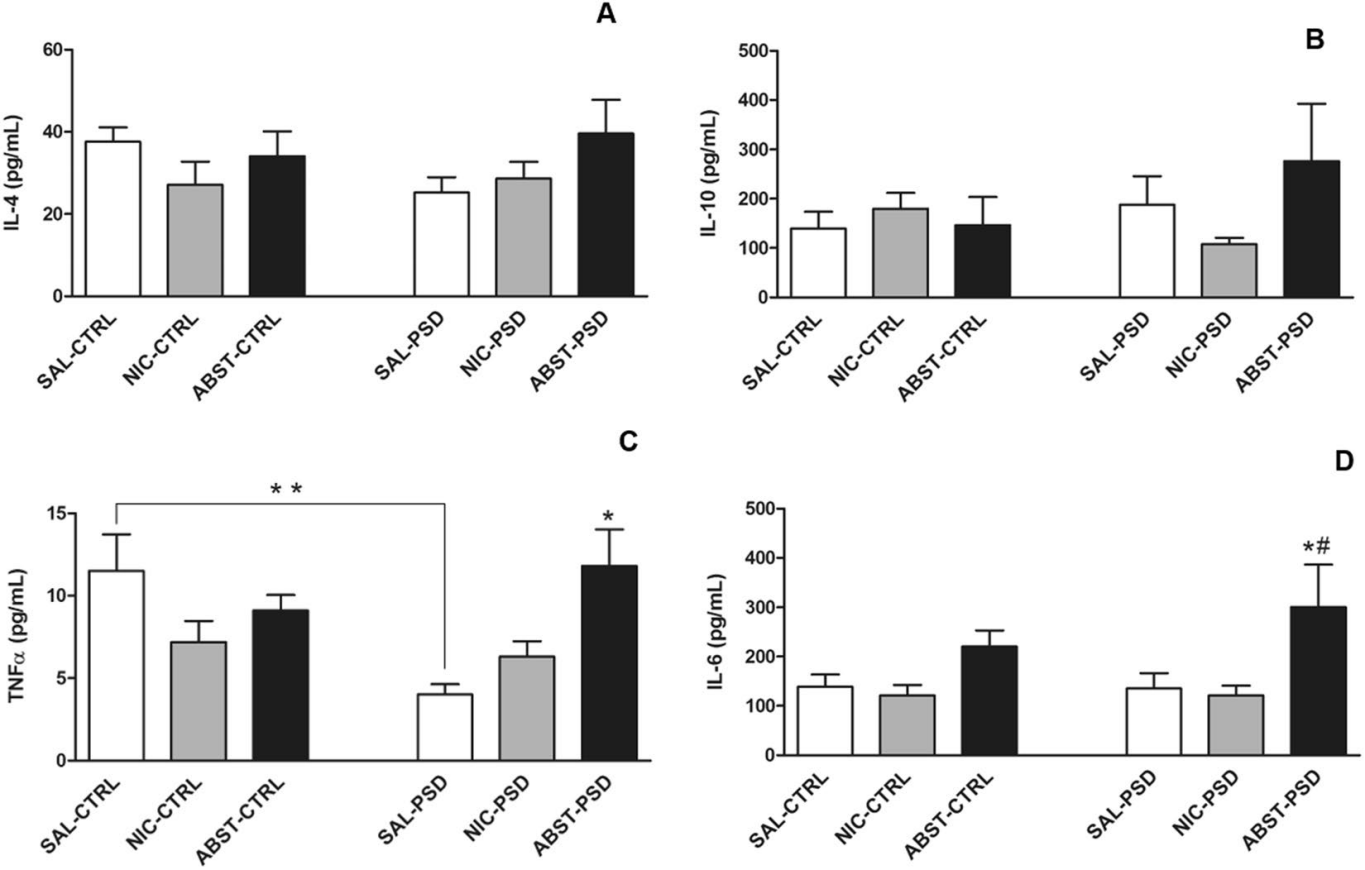
Plasmatic cytokines. Mean ± standard deviation of anti-inflammatory cytokines interleukin (IL)-4 (pg/mL, **A**) and IL-10 (pg/mL, **B**) and, pro-inflammatory cytokines tumor necrosis factor (TNF)-α (pg/mL, **C**) and IL-6 (pg/mL, **D**) in plasma samples from animals submitted to a 12-day saline (SAL) treatment, a 12-day nicotine (NIC, 3.2 mg/kg/day) treatment or a 7-day NIC treatment followed by mecamylamine-induced withdrawal (1.5 mg/kg) and subsequently exposed to control sleep condition (CTRL) or 72 h paradoxical sleep deprivation (PSD) (n = 10 per group). *p < 0.05 compared to SAL-PSD; ^#^p < 0.05 compared to NIC-PSD.

No significant effects were observed in the plasmatic concentrations of the anti-inflammatory cytokines IL-4 and IL-10. (Fig. 4A,B). However, there was a significant interaction effect on plasmatic TNF-α concentrations (F_2,54_ = 5.1, p < 0.01); and treatment (F_2,54_ = 6.3, p < 0.01) and interaction (F_2,54_ = 6.1, p < 0.05) effects on IL-6 concentrations. *Post-hoc* tests showed that the ABST-PSD group presented higher concentrations of TNF-α compared to SAL-PSD (p = 0.001), which in turn showed lower concentrations compared to the SAL-CTRL (p = 0.001) group (Fig. 4C). Overall, higher levels of IL-6 were found in the ABST animals compared to both SAL (p = 0.001) and NIC groups (p < 0.0001). *Post-hoc* analysis of the interaction effect revealed, however, that this increase in IL-6 levels occurred specifically in the ABST-PSD group compared to SAL- SAL-PSD (p = 0.020) and NIC-PSD (p = 0.006) as well as compared with CTRL (p = 0.026) and NIC-CTRL (p = 0.006) groups (Fig. 4D). No other differences were observed between groups.

### Nicotine Treatment, Sleep Deprivation, and IL-4 as Predictors of Pain Sensitivity

A correlation matrix between the latency to paw withdrawal, the delta body weight and the immunological parameters was calculated for the whole sample to reveal possible factors associated with pain threshold (Table 1).

**Table 1:** Correlation of latency to paw withdrawal with weight loss and plasmatic concentrations of cytokines (n = 60).

	R	p
Δ Body weigth	0.58	<**0**.**0001**
IL-4	0.26	<**0**.**05**
IL-6	−0.01	>0.05
IL-10	−0.07	>0.05
TNF-α	0.14	>0.05

Δ: Delta (difference between final and initial); IL: interleukin; TNF-α: tumor necrosis factor alfa; R: Pearson correlation coefficient; p: statistical significance.

Then, a generalized linear model was fitted considering the latency to paw withdrawal as the dependent variable (Table 2). The best model included PSD (Wald: 15.7, df: 1, p < 0.0001), treatment (Wald: 24.5, df: 1, p < 0.0001) and IL-4 (Wald: 5.9, df: 1, p < 0.05) as the independent predictors of the latency to paw withdrawal (Likelihood ratio χ^2^ = 96.3, df: 9, p < 0.0001, n = 60). An increase in 10 pg/mL of plasmatic IL-4 corresponded to an average increase of 6% in the latency to paw withdrawal, thus revealing an association between higher plasmatic concentrations of IL-4 and a higher pain threshold. Additionally, PSD was associated with an average decrease of 49.9% in the latency to paw withdrawal compared to CTRL condition, while repeated nicotine administration was associated with an average increase of 63.8% in comparison with saline administration, as shown in Table 2.

**Table 2:** Statistically significant predictors of paw withdrawal latency resulting from the generalized linear model (*tweedie* regression, n = 60).

	OR	95% CI	χ^2^	p
(Constant)	—	14.091–27.228	313.384	**<0.0001**
Sleep				
*PSD*	0.501	0.356–0.705	15.674	**<0.0001**
*CTRL*		Reference		
Treatment				
*Nicotine*	1.638	1.311–2.046	18.922	**<0.0001**
Mecamylamine*	0.953	0.796–1.142	0.272	>0.05
*Saline*		Reference		
IL-4	1.006	1.001–1.010	5.848	**<0.05**

CI: confidence interval; IL-4: interleukin 4; OR: odds ratio; PSD: paradoxical sleep deprivation; χ^2^: Wald chi-square value; *refers to the protocol of nicotine withdrawal.

Within SAL-CTRL and NIC-CTRL groups, it was observed a moderate positive correlation between paw withdrawal latency and IL-4 levels (Supplementary Table 1). A trend (p = 0.07) was also found in the ABST-CTRL group, which showed a positive correlation of IL-6 and IL-10 levels with paw withdrawal latency (Supplementary Table 1). Body weight loss was only correlated with paw withdrawal latency in the SAL-PSD group (Supplementary Table 1).

## Discussion

Our results have shown that both nicotine and PSD independently affected thermal pain sensitivity, leading either to a decrease or to an increase in paw withdrawal latency, respectively. When associated, the pronociceptive effects of PSD prevailed over the antinociceptive effects of nicotine treatment, with PSD animals displaying hyperalgesia regardless of previous repeated nicotine administration. PSD combined with nicotine abstinence synergistically increased the plasmatic concentrations of IL-6. Additionally, when body weight loss, TNF-α, IL-4 and IL-6 levels were included in the model as confounders, ABST-PSD animals showed the lowest pain threshold compared to all groups, with no significant difference compared to the SAL-PSD group. The regression model demonstrated that repeated nicotine administration and higher concentrations of IL-4 were independent predictors of higher thermal pain threshold, while PSD was associated with a 50% decrease in pain threshold in the hot plate test.

As part of the mesolimbic dopaminergic pathway, nucleus accumbens seems to mediate the reinforcing and aversive effects of nicotine and its withdrawal^16,17^, as well as to play a role in pain modulation^18,19^. Nicotine has been shown to increase synaptic dopamine and D2 receptor sensitivity in the nucleus accumbens^20–22^. Both D1 and D2 dopamine receptors are expressed by neurons in the ventrolateral periaqueductal gray (vlPAG), playing a central role in morphine and dopamine-induced antinociception^23,24^. The vlPAG act as an output system responsible for integrating afferent inputs from multiple forebrain areas to modulate nociception at the spinal dorsal horn^25^. Recently, Umana *et al*. has shown that 63% of the projections from vlPAG to the rostral ventromedial medulla express α7 nicotinic acetylcholine receptors (nAChR), suggesting a model of α7 nAChR-mediated analgesia in the vlPAG^26^. In their study, systemic and intra-vlPAG administration of an α7 nAChR-selective agonist showed an antinociceptive effect in the formalin assay, which was blocked by intra-vlPAG α7 antagonist pretreatment^27^. Nicotine administration increased GABAergic transmission via presynaptic nAChRs of both α7-lacking and α7-expressing neurons in the vlPAG of rats^27^. Previous evidence indicates that nicotine might exert its antinociceptive effect mainly through modulation of α4β2 nAChRs^6,28,29^. Activation of α4β2 nAChRs results in stimulation of dorsal raphe, nucleus raphe magnus, and locus coeruleus in a norepinephrine (NE)-dependent fashion^27–30^. These areas play a significant role in pain modulation through descending inhibitory pathways, underlying in part the nicotine-induced antinociception^31^. In addition, systemic administration of nicotine significantly increases the release of endogenous opioids such as endorphins, enkephalins, and dynorphins in the supraspinal cord via α7 nAChR^30^.

Of note, vlPAG descending pathway seems to be the key site by which PSD increases nociceptive responses in rats. A recent study showed that PSD decreased morphine-induced analgesia by modulation of the vlPAG in rats^31^. Moreover, Sardi and colleagues^27^ have shown that the nucleus accumbens also mediates the pronociceptive effect of PSD through activation of A2A adenosine receptors and inhibition of D2 dopamine receptors. In this study, an excitotoxic lesion of the nucleus accumbens prevented the PSD-induced hyperalgesia, which was reverted by an acute blockade of this region through either an A2A adenosine antagonist or a D2 dopamine agonist^27^. Considering that A2A receptors are also largely involved in the homeostatic regulation of the sleep-wake cycle^32^, we might speculate that the role of nucleus accumbens A2A receptors in the pronociceptive effect of PSD would also be linked to sleep pressure. The greater the sleep need, the greater the A2A receptors activity, and, thus, the lower the pain threshold^27^.

Sleep deprivation has been shown to decrease D2 receptor expression in the nucleus accumbens of humans^33^ but to increase its sensitivity in rats subjected to 96 h of PSD^34^, though it has been shown that freely moving rats exhibit higher concentrations of dopamine in the nucleus accumbens during REM sleep^35^. Considering that vlPAG receives projections from nucleus accumbens neurons expressing A2A receptors, a possible integrative mechanism for the pronociceptive effects of PSD could rely on the increased nucleus accumbens A2A activity, leading to an activation of the vlPAG descending pathway. Even though there is no study assessing a role of nucleus accumbens adenosine receptors in pain processing, the use of adenosine receptor agonists has shown potent antinociceptive effects in animal models of chronic pain^36^. Spinal cord neurons expressing A2A adenosine receptors seems to mediate antinociception, inhibiting symptoms of neuropathic pain^37^. On the other hand, theophylline (an adenosine receptor antagonist) reduced antinociception induced by nicotine in the formalin test^38^. Taken together the evidence from the literature, we can speculate that in our study PSD overcame the antinociceptive effects of nicotine pretreatment by inhibiting the pain inhibitory descending vlPAG pathway through A2A receptor activation and D2 receptor inhibition in the nucleus accumbens^27^. Possibly, the antinociceptive effects of nicotine involved multiple pathways, including activation of α4β2 nAChRs^29^ and of spinal A2A receptors^36^, release of opioids at the spinal cord^30^ as well as nucleus accumbens activation with projections to vlPAG through α7 nAChR^26^.

Pro-inflammatory cytokines, such as IL-6 and TNF-α, exert known pro-nociceptive effects through central and peripheral action and are implied in the pathophysiology of neuropathic pain^39–42^. We observed an increase in plasmatic concentrations of IL-6 when nicotine abstinence and PSD were associated. Nonetheless, there was no correlation between pain threshold and the cytokines in the ABST-PSD group possibly due to the strong pro-nociceptive effect of PSD itself, leading to a ceiling effect. However, when we adjusted the analysis of pain threshold for delta body weight, IL-6, IL-4 and TNF-α, the ABST-PSD group showed the lowest latency to paw withdrawal, suggesting a synergic effect of nicotine withdrawal and PSD. Our findings point towards an inflammatory component in thermal sensitivity, as IL-4 was a predictor associated with pain threshold, although it did not differ statistically between the groups. With the observed OR of 1.006 and the mean concentration of IL-4 = 32.04 pg/mL, IL-4 may account on average for 21.13% of the thermal pain threshold variability in our sample. Thus, IL-4 may partially explain endogenous spontaneous individual differences in pain threshold, independent of nicotine treatment or of PSD. Animal studies have shown that both IL-4 and IL-10 exert an antinociceptive effect in various models of inflammatory pain; however, both cytokines were unable to increase pain threshold in control animals^43–45^. Although IL-4 up-regulates the expression of opioid receptors, the opioid antagonist naloxone did not reverse the antinociceptive effect of IL-4 in a model of inflammatory pain^45^. In addition, IL-4 knockout mice did not demonstrate a lower latency in the hot plate test, yet showed a lower pain threshold in the von-Frey test^46^. This discrepancy of results might be explained by different species used and different physiological conditions, as IL-4 seems to exert an antinociceptive effect more prominently in hyperalgesic conditions. From a translational point of view, a clinical study has found lower proteic and mRNA expression of IL-4 and IL-10 in patients with chronic pain^47^.

PSD is known to cause a low-grade inflammation in rats primarily through an elevation of the cytokines IL-1β, IL-6, and TNF-α compared to controls^48–51^. However, data from the literature is contradictory, possibly due to differences in sleep deprivation models, protocol specificities, and different animal strains used. In our study, 72 h of PSD did not have an independent effect on TNF-α and IL-6 levels. On the contrary, the SAL-PSD group showed a decrease in the TNF-α levels compared to the SAL-CTRL group, contributing to the statistical difference found between ABST-PSD and SAL-PSD. We should consider that our study design involved chronic daily subcutaneous injections (twice a day), which is not the same as having naïve animals. All animals displayed similar high levels of corticosterone at the end of the protocol (data not shown), indicating that the daily injections were possibly stressful to the animals and should be considered in the interpretation of the data. Taking into consideration the possible effect of stress in a context of a pro-inflammatory stimulus, we found evidence from the literature that may help explain the unexpected finding of TNF-α. Rats exposed to heat stress or to sodium arsenite 18 h prior to lipopolysaccharide (LPS) administration, a well-known pro-inflammatory stimulus, had significantly lower levels of plasma TNF-α – instead of higher – leading to a decreased mortality and lung injury^52^. This finding suggests a protective or preconditioning effect of stress response before a pro-inflammatory stimulus. Thus, we could speculate that the stress involved in the subcutaneous daily injections altered the expected pro-inflammatory effect of PSD in saline-treated animals as a preconditioning stimulus.

Nicotine, on the contrary, exerts an anti-inflammatory effect in animal models, decreasing the levels of pro-inflammatory cytokines, namely, IL-1β, IL-6, TNF-α, and IL-17^13,14,53^. In our study, the lack of statistical differences in plasma concentrations of pro-inflammatory and anti-inflammatory cytokines between NIC-CTRL and SAL-CTRL groups may be due to the lack of an injury stimulus, since the previous studies in which nicotine showed an anti-inflammatory action were performed in different contexts, such as LPS administration, virus infection, and lung injury^13,14,53^. The anti-inflammatory effects of nicotine seem to be modulated by the cholinergic anti-inflammatory pathway^15^, mainly through α7 nAChRs in immune cells. Prolonged exposure to nicotine inactivates both α7 and α4β2 nAChRs^54–56^. Thus, another possibility could be the development of tolerance to the anti-inflammatory effects of nicotine via α7 desensitization induced by the chronic nicotine treatment, contributing to the effect of nicotine abstinence.

Our study indicates that nicotine abstinence combined with PSD may synergistically increase IL-6 levels. Vagal tone is decreased in sleep-deprived rats^57^, which, in addition to desensitization of α7 receptors in response to repeated nicotine administration, might explain the synergistic interaction effect of nicotine withdrawal and PSD on plasmatic concentrations of IL-6. Our data suggest that, during nicotine abstinence, sleep deprivation may predispose the organism to inflammation.

It is important to consider the limitations of the current study. Although we followed a very consistent and standardized protocol of mecamylamine-induced abstinence, we did not assess the behavioral signs of nicotine withdrawal in the animals. We recognize that the use of additional tests based on mechanical or chemical pain sensitivity could avoid a ceiling effect of PSD in the pain threshold assessment, and possibly allow a further understanding of the interactions between PSD and nicotine administration/withdrawal. Also, the concentrations were determined in animals that had undergone the hot plate test and an effect of test exposure on cytokine concentrations should not be ruled out. Additionally, central nervous systems cytokine concentrations could yield additional information about neural pathways underlying the interaction between PSD and nicotine and its effects on nociception. Lastly, we did not include a reinstatement group of nicotine after its withdrawal.

## Conclusion

Our study confirmed the previously observed effects of nicotine and PSD on nociception and showed that the PSD-induced pronociceptive effects largely prevailed over nicotine-induced antinociception. When associated with PSD, however, nicotine abstinence synergistically increased IL-6 levels and independently decreased pain threshold. Higher levels of IL-4 were independently associated with higher pain threshold.

## Methods

### Animals

Sixty 12-week-old male Wistar rats were obtained from *Centro de Desenvolvimento de Modelos Experimentais para Medicina e Biologia* (CEDEME, Universidade Federal de São Paulo, São Paulo, Brazil). All experiments were approved by the Animal Ethics Committee of Universidade Federal de São Paulo (#742480/2013) and followed international ethical standards^58^, complying with the National Institutes of Health Guide for the Care and Use of Laboratory Animals (8^th^ Edition, revised 2011) and the recommendations of the American Association for Accreditation of Laboratory Animal Care. Animals were maintained in groups of five per cage in individually ventilated cages (Tecniplast, Italy) in a temperature-controlled room (23 ± 1 °C) with a 12:12 h light-dark cycle (lights off at 19 h). Food and water were provided *ad libitum* throughout the protocol.

### Drugs and Treatment

Nicotine-treated animals received 3.2 mg/kg/day of nicotine (nicotine hydrogen tartrate, Sigma, USA) in 2 daily subcutaneous (s.c.) injections for 12 days. This dose has been known to reliably induce nicotine dependence^59–61^. In order to establish nicotine abstinence, rats were first sensitized to nicotine during 7 days, a period that has been shown to be sufficient to induce sensitization to nicotine in adult rats^62,63^. Animals in the abstinence group were then acutely treated with mecamylamine hydrochloride (2-methylaminoisocamphane hydrochloride, Sigma, USA) on day 8 at 7 h and 19 h (1.5 mg/kg, s.c.). For the remainder of the days (days 9 through 12) animals in the abstinence groups received solely saline injections twice a day (s.c.). Mecamylamine is a non-competitive nicotinic cholinergic receptor antagonist that precipitates symptoms of nicotine abstinence in sensitized rats 1 day after its administration^64^. A previous study demonstrated significant effects of mecamylamine dose on intracranial self-stimulation thresholds and total somatic signs (withdrawal-like signs), with significant increases in both measures at the 1.5 mg/kg dose^64^. The nicotine dose chosen for inducing abstinence symptoms upon mecamylamine treatment has been previously described elsewhere^64,65^.

All drugs were diluted in sterile physiologic saline (0.9%) and had their pH corrected for 7.4. Subcutaneous injections were always administered in 1.0 mL/kg volume. Animals from the sham groups were treated with saline similarly to the other groups.

### Paradoxical Sleep Deprivation

Animals were submitted to 72 h of PSD using the modified multiple platform method^66^. Groups of 5 animals were housed in a water-filled tank (143 × 41 × 30 cm) containing 12 circular platforms (6.5 cm in diameter), whose surface was 1 cm above the water level. Rats could move jumping from one platform to another. When animals reach paradoxical sleep phase, they experience loss of muscle tone and fall into the water, being awakened. Groups of 5 sleep control animals were housed in home cages out of ventilated racks in the same room as the PSD animals during this protocol. We elected to choose neither a too long (96 h) nor a too short period (24 h) of PSD. However, the literature is not consistent about the effects of 48 h of PSD on thermal pain sensitivity^67^. Asakura and colleagues did not find significant differences in the latency of paw withdrawal in the hot plate test after 48 h of PSD. With regard to 72 h of PSD, however, most of the studies showed a significant hyperalgesic effect in thermal pain sensitivity^9,68^. Thus, we chose 72 h of PSD since it would certainly lead to hyperalgesia in the hot plate test and not be considered ethically aggressive as 96 h of PSD.

### Nociceptive Evaluation

Thermal pain sensitivity was evaluated using the hot plate test^69^ in a protocol previously used in sleep-deprived animals^9,70^. During the test, each animal was individually placed in a 50 °C-heated hot plate apparatus, and the latency to paw withdrawal was measured as an estimation of pain threshold. At the first sign of paw withdrawal, i.e., a behavior of paw licking or jumping as an attempt to escape, the rat was removed from the hot plate. The test had a maximum duration of 90 s to avoid paw lesions and burns. The hot plate was cleaned with ethanol 30% between each test.

### Sample Collection

At the end of the experimental protocol (Day 12), rats were rapidly decapitated with minimum discomfort after the hot plate test. The euthanasia schedule was standardized at 8 h. Blood was collected into sterile tubes with liquid EDTA and centrifuged at 4 °C and 1300 *g* for 10 min to obtain separated plasma. Plasma samples were frozen at −20 °C for further analyses.

### Cytokines Concentrations

For cytokine quantification, the Luminex® platform (Millipore, USA) was used following manufacturer’s instructions. Milliplex® Map kits (*Rat Cytokine/Chemokine Panel*) were used to determine the plasma concentration of interleukin (IL)-4, IL-6, IL-8, IL-10, and tumor necrosis factor (TNF)-α. Briefly, each cytokine binds to its specific antibody-coated microsphere, which contains 2 fluorochromes. This combination of fluorochromes allows for the determination of which cytokine is bound to each microsphere. Additionally, another antibody binds to the cytokine-microsphere complex, thus determining the concentration of each cytokine.

### Statistical Analysis

All continuous data were firstly tested for normality and homogeneity. Variables without normal distribution or homogeneity among the groups were standardized by z-score. Between-group comparisons were performed using 2-way analysis of variance (ANOVA) test considering treatment (SAL, NIC or ABST) and sleep condition (CTRL or PSD) as independent variables. For treatment effect or interaction effect, the statistical difference was calculated using Bonferroni *post-hoc*. Body weight at the beginning and the end of the protocol was compared by repeated measures 2-way ANOVA, followed by Bonferroni’s *post-hoc* if necessary. To further understand the independent effects of treatment, sleep condition and its interactions, multiple analysis of covariance (MANCOVA) was performed for latency to paw withdrawal (dependent variable) with adjustment for the potential covariates (delta body weight, TNF-α, IL-4 and IL-6). Pairwise comparisons were performed by Bonferroni’s *post-hoc*. Correlations between continuous data were calculated through Pearson’s correlation test. Finally, a generalized linear model with *tweedie* distribution and log link function was applied to establish predictor variables for pain sensitivity using latency to paw withdrawal as the dependent variable and body weight variation, treatment, sleep deprivation, and cytokines as the independent variables. For statistical significance, we adopted α = 0.05.

## Electronic supplementary material

Table 1
