# Supplementary material for: Nicotine and sleep deprivation: impact on pain sensitivity and immune modulation in rats
Source: Sci Rep. 2018 Sep 14;8:13837. doi: 10.1038/s41598-018-32276-7 (PMC6138689; doi:10.1038/s41598-018-32276-7)
Supplement: Supplementary file 1 — Table 1 [file 41598_2018_32276_MOESM1_ESM.doc]

**Nicotine and sleep deprivation: impact on pain sensitivity and immune modulation in rats**

Camila Hirotsu^1^*, Matheus Negrão Pedroni^1^*, Laís Fernanda Berro^2^, Sergio Tufik^1^ and Monica Levy Andersen^1^

^1^Department of Psychobiology, Universidade Federal de São Paulo, São Paulo, Brazil

^2^Department of Psychiatry and Human Behavior, University of Mississippi Medical Center

*Both authors contributed equally to this study

**Supplementary Table 1.** Correlation between paw withdrawal latency and metabolic and immunological parameters within each experimental group (n=10/group).

|  |  | **IL-4** | **IL-1α** | **IL-6** | **IL-10** | **TNF-α** | **Δ Body weigth** |
| --- | --- | --- | --- | --- | --- | --- | --- |
| **SAL+CTRL** | R | **0.67** | 0.53 | 0.13 | -0.53 | 0.35 | 0.15 |
|  | p | **0.03** | 0.12 | 0.72 | 0.12 | 0.33 | 0.68 |
| **SAL+PSD** | R | 0.02 | 0.08 | -0.16 | 0.13 | -0.13 | **0.71** |
|  | p | 0.95 | 0.82 | 0.65 | 0.72 | 0.71 | **0.02** |
| **NIC+CTRL** | R | **0.66** | 0.40 | **0.72** | -0.46 | 0.38 | -0.09 |
|  | p | **0.04** | 0.26 | **0.02** | 0.18 | 0.28 | 0.80 |
| **NIC+PSD** | R | 0.51 | 0.09 | -0.11 | -0.22 | 0.16 | -0.20 |
|  | p | 0.13 | 0.81 | 0.76 | 0.54 | 0.65 | 0.59 |
| **ABST+CTRL** | R | 0.59 | 0.39 | **0.64** | **0.66** | 0.41 | -0.23 |
|  | p | 0.07 | 0.27 | **0.05** | **0.04** | 0.24 | 0.52 |
| **ABST+PSD** | R | 0.42 | 0.50 | 0.09 | 0.19 | 0.08 | 0.27 |
|  | p | 0.23 | 0.14 | 0.81 | 0.60 | 0.83 | 0.45 |

Δ: difference between initial and final; IL: interleukin; TNF-α: tumor necrosis factor; R: Pearson correlation coefficient; p: statistical significance.
